# Predicting Relevant Microglia‐Associated Cell–Cell Communication Pathways in Alzheimer's Disease: A Role for SPP1

**DOI:** 10.1002/glia.70210

**Published:** 2026-08-04

**Authors:** Janssen M. Kotah, Marina Trombetta Lima, Esmée C. Dragt, Asimenia Voulgaroglou, Nieske Brouwer, Inge R. Holtman, Susanne M. Kooistra, Bart J. L. Eggen

**Affiliations:** ^1^ Department of Biomedical Sciences University of Groningen, University Medical Center Groningen Groningen the Netherlands

**Keywords:** Alzheimer's disease, cell–cell communication, microglia, single nucleus RNA sequencing, SPP1

## Abstract

Microglia play a key role in the pathophysiology of Alzheimer's Disease (AD) and their increased heterogeneity likely affects disease progression. We previously identified distinct microglial signatures that were enriched in AD donors and associated with amyloid and tau, respectively. Here we generated a snRNAseq dataset from postmortem control and AD cases and analyzed alterations in cell–cell communication pathways that might be relevant to AD pathophysiology. One signaling pathway perturbed in AD cases involved *SPP1*, and while this pathway was also present in control samples, microglia–microglia SPP1 signaling was restricted to AD donors. Further analyses within microglia–microglia signaling predict AD‐specific induction of *GAS6‐AXL* signaling (from inflammatory and ribosomal microglia), and *SPP1‐ITGAV/ITGB5* signaling (from disease‐associated and inflammatory microglia, among others). Together, these findings might in part explain the increased microglia phagocytic profile described in AD. RNAscope confirmed enrichment of *SPP1* expressing microglia near amyloid plaques in AD brain tissue samples. These data indicate altered cellular communications between microglia in the AD brain.

## Graphical abstract

- We used CellChat to infer altered cell–cell communication in Alzheimer's disease
- Microglia signal to each other via SPP1 in Alzheimer's disease.
- *SPP1‐*expressing microglia are present around amyloid‐β plaques.

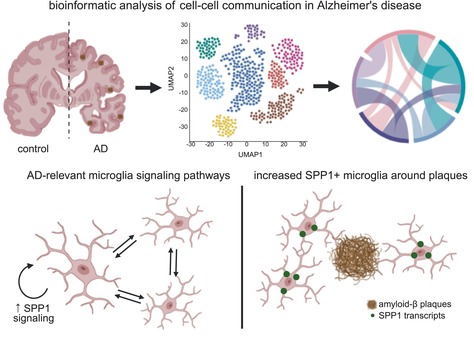

## 1 Introduction

Alzheimer's disease (AD) is the most prevalent form of dementia, characterized by neuropathological aggregation of amyloid‐beta (Aβ) plaques and neurofibrillary tau tangles (Knopman et al. 2021). Work over the past two decades has highlighted the important role of neuroinflammation in AD pathophysiology (Brown et al. 2026; Butovsky et al. 2025; Heneka et al. 2025). Microglia, the resident immune cells in the brain parenchyma (Paolicelli et al. 2022), are implicated in AD, as further suggested by their enriched expression of AD risk genes identified via genome‐wide association (Karch and Goate 2015; Sudwarts and Thinakaran 2023; Nott et al. 2019). Microglia also respond strongly to both amyloid‐beta (Aβ) plaques (Heneka et al. 2015), and tau pathology (Falkon et al. 2025; Wang et al. 2022). The phagocytosis and neuroinflammation induced in microglia by these pathological features likely result in their contribution to the neurodegeneration seen in AD (Gao et al. 2023; Taddei et al. 2023).

Advancements in (single cell) transcriptomic technologies have helped to better understand how microglia respond to and contribute to neurodegenerative diseases such as AD. Most of this work involved characterizing the spectrum of microglial transcriptional states. This includes work performed on mouse models of amyloidosis (Keren‐Shaul et al. 2017; Krasemann et al. 2017), but also work from postmortem human brain samples (Mathys et al. 2019, 2023; Srinivasan et al. 2020; Sun et al. 2023), which has resulted in the identification of gene signatures enriched in microglia derived from diseased donors. Our group previously contributed to this by performing single‐nucleus RNA sequencing (snRNAseq) on frozen postmortem samples enriched for non‐neuronal and non‐oligodendrocyte lineage nuclei using fluorescence activated nuclear sorting (Gerrits et al. 2021). Two distinct microglial states were identified, whose relative abundance correlated with the extent of Aβ and tau pathology, respectively. These microglial states might be expected to occupy different niches (van Olst et al. 2025), and differentially interact with the local microenvironment they are in. This includes how they communicate with other cells in the CNS, which can be bioinformatically inferred based on high dimensional snRNAseq data (Almet et al. 2021; Jin et al. 2021, 2025). However, because of the glial enrichment performed in the original study, we could not properly interrogate how microglia‐associated cell–cell interactions are affected in disease.

To this end, we supplemented our original snRNAseq dataset (Gerrits et al. 2021) with newly generated neuron‐ and oligodendrocyte‐enriched snRNAseq data and used bioinformatic tools to predict cell–cell interactions. After snRNAseq data integration, CellChat (Jin et al. 2021, 2025) was used to predict changes in cell–cell communication, and multiple signaling pathways were predicted to be altered in AD donors. In a separate set of control and AD tissue samples, RNAscope and immunofluorescence validations confirmed increased microglial expression of a component of one of these pathways, which involved *SPP1*.

## 2 Methods

### 2.1 Tissue Selection

For snRNAseq, postmortem human brain samples were obtained from the NeuroBiobank of the Institute Born‐Bunge (NBB‐IBB), Wilrijk (Antwerp), Belgium (ID: BB190113), who previously obtained informed consent from the donors to donate their brain. Ethical approval was granted by the medical ethics committee of the Hospital Network Antwerp (ZNA, approval numbers 2805 and 2806). We used 10 (5 CTR, 5 AD) fresh frozen brain tissue samples from the occipitotemporal cortex. These samples had previously been used to generate a glia enriched snRNAseq dataset (Gerrits et al. 2021). For RNAscope analysis, we obtained 12 (4 control, 8 AD) formalin‐fixed, paraffin embedded (FFPE) superior parietal gyrus brain tissue samples from the Netherlands Brain Bank (NBB, Amsterdam, the Netherlands, https://www.brainbank.nl). The Netherlands Brain Bank had previously obtained ethical approval from the ethical committee of the VU University Medical Center in Amsterdam, the Netherlands (reference number 2009/148), as well as prior written consent from donors for the use of material and clinical data for research purposes.

### 2.2 Nuclear Isolation and Preparation of Single Nucleus Libraries

Nuclei were isolated from fresh frozen brain samples as previously described (Gerrits et al. 2021). Briefly, approximately 10 cryostat sections of 40 μm thickness were lysed in a sucrose lysis buffer (10 mM Tris–HCL (pH 8.0); 0.32 M sucrose; 5 mM CaCl2; 3 mM Mg(Ac)2; 0.1 mM EDTA; 1 mM dithiothreitol (DTT) and 0.1% Triton X‐100). After homogenization by Dounce pottering in lysis buffer, the suspension was filtered through a 70 μm cell strainer (Falcon, Corning Inc., USA) and layered on a 1.8 M sucrose gradient (10 mM Tris–HCL (pH 8.0); 3 mM Mg(Ac)2; 0.1 mM EDTA and 1 mM DTT). Nuclei were purified by ultracentrifugation at 107,000 × *g* for 90 min at 4°C using an Optima L‐100XP Ultracentrifuge equipped with a SW55Ti swinging‐bucket rotor (Beckman Coulter Life Sciences, USA). Nuclear pellets were suspended in 2% BSA/PBS containing 0.35 U/mL RNase inhibitor (#EO0381 ThermoFisher Scientific, USA) and incubated with fluorescently conjugated antibodies against NEUN (RBFOX3/NEUN (1B7) AF647 mouse mAB, Novus Biologicals, NBP1‐92693AF647) and OLIG2 (clone 211F1.1 AF488 mouse mAb, Merck Millipore, MABN50A4). After washing and adding DAPI, nuclei were sorted on a MoFlo Astrios (Beckman Coulter Life Sciences, USA). For each sample, we collected 30,000 NEUN+ nuclei, 15,000 OLIG2+ nuclei, and 5000 NEUN‐/OLIG2‐nuclei. Samples were kept on ice throughout the isolation and staining procedure. Single nucleus libraries were constructed using the Chromium Single Cell 3′ Reagents Kit v3.1 and corresponding user guide (10× Genomics). Samples were pooled in equimolar ratios and sequenced on a NextSeq 500 (v2.5) at the Research Sequencing Facility of the UMCG.

### 2.3 snRNA Analysis Pipeline

After aligning raw sequencing reads to the GRCh38‐2020‐A reference genome using CellRanger 7.0.0, subsequent bioinformatic analyses were performed in Seurat v4.3.0, on either R v4.2.3 (local analyses) or v4.2.1 (analyses performed on a high‐performance cluster). Prior to Seurat analyses, aligned counts were first adjusted for ambient RNA contamination using SoupX v1.6.2 (Young and Behjati 2020) with default settings, and potential doublets were identified using Scrublet v0.2.3 (Wolock et al. 2019), with an expected doublet rate of 0.1. Each library was then loaded into Seurat for pre‐processing, filtering for genes expressed in a minimum of 3 nuclei, and filtering for nuclei with at least 200 transcripts detected. Nuclei with more than 5% ribosomal or mitochondrial percentage were then filtered out. To jointly analyze libraries from multiple samples together, we merged individual Seurat objects, log‐normalized counts, and selected variable features that were used to compute principal component embeddings. For clustering analysis involving multiple cell types, the top 2000 variable features were used, which were then scaled using the ScaleData function in Seurat, regressing out total RNA counts and percentage mitochondrial gene expression. When subclustering within a cell type (e.g., within neurons), we used the top 4000 variable features to calculate principal components, and regressed out total RNA count, mitochondrial content, and ribosomal content. PCA embeddings were harmonized using the Harmony algorithm (Korsunsky et al. 2019), using “sample” as the variable to integrate. After constructing nearest‐neighbors graph, UMAP embeddings were computed using the RunUMAP function, and clusters were identified using the FindClusters function using the Louvain algorithm. Across rounds of cell (sub)type, we calculated 40 principal components, which were then all subject to harmonization.

The number of harmonized components used to construct the nearest‐neighbor graph and calculate the UMAP embeddings varied by cell type. To make a consistent measure of the number of harmonized principal components, we made use of the recommendation by the Harvard Chan Bioinformatics Core (https://github.com/hbctraining/scRNA‐seq). In brief, we calculated two metrics: the first metric involved calculating the percent variation associated with each harmonized principal component and then calculating the cumulative percent for each harmonized principal component. The component of interest from this metric is the first component, explaining less than 5% of variation on its own, for which the cumulative percentage reaches 90. The second metric is based on identifying the first component in which the difference in percent variation accounted for compared to the previous component is less than 0.1%. The minimum of both metrics is then used as the limit for the number of harmonized principal components used as input for subsequent analyses.

As differences in CellRanger versions have recently been demonstrated to have an effect on downstream analyses (Abugessaisa et al. 2024), we re‐aligned the sequencing reads from the (Gerrits et al. 2021) dataset, which was previously analyzed on CellRanger v3, before including them in our analyses. Downstream analyses, including integration to the neuron‐ and oligodendrocyte‐enriched dataset generated in this manuscript, were performed as outlined above. When integrating both datasets, samples were integrated based on a Sample × Dataset interaction term in the metadata to account for the fact that samples were shared between datasets.

Cell type annotation was performed based on finding marker genes (using the FindAllMarkers function), and cross‐ referencing enriched genes to expected cell types from reports in literature. (Sub)clustering analyses were performed over several rounds and were done with a focus on first removing clusters that might represent bad quality cells (e.g., based on being enriched in predicted doublets/having high doublet scores, aberrant number of total/unique/mitochondrial/ribosomal genes, and co‐expression of genes associated with distinct cell types). After this was done, we then attempted to annotate sub‐types of cells, when possible, based on previously described literature. For excitatory neurons, we expected molecular diversity to reflect cortical layering, and thus utilized a previously reported spatial transcriptomics dataset in cortical samples (Maynard et al. 2021). The top 100 positive markers per excitatory neuronal cluster was treated as a gene set, for which we calculated either a module score per spatial spot in the Maynard dataset, and subsequently plotted in space. Separately, we also utilized the Shiny web‐app presented in the Maynard paper to calculate a gene set enrichment for each set of cluster markers to obtain a more quantitative metric for which cortical layers our clusters corresponded to. For inhibitory neurons, we used available atlases (Hodge et al. 2019; Wei et al. 2022) to stratify the two major subgroups of medial or caudal ganglionic eminence interneurons. For microglia, we annotated microglial states using both marker gene expression and signatures previously reported in literature (Mancuso et al. 2024).

Cell (sub)type proportion analyses within the excitatory and inhibitory neuronal groups were performed using propeller function in the speckle package v1.2.0 (Phipson et al. 2022), by creating a linear model that quantified the impact of diagnosis on a cluster's relative abundance, whereas correcting for sex, age, and postmortem delay. Cell–cell communications were inferred using the CellChat package v2.2.0.9001 (Jin et al. 2021, 2025). Briefly, the total, integrated Seurat objects were split into two based on diagnosis. Total cellular interactions were then separately calculated within the control and AD subsets using the CellChatDB database (non‐protein signaling annotations removed). We used the differences between the scores for each interaction pair between control and AD as evidence for enrichment or depletion of specific signaling pathways in disease. Because we identified unique, AD‐associated microglia–microglia interactions in this analysis, we repeated the CellChat analysis on a subset of the data in which only microglia were present.

### 2.4 RNAscope In Situ Hybridization

#### 2.4.1 Tissue Processing

We combined RNAScope in situ hybridization and immunofluorescence on 5 um sections of tissue, cut from a microtome, in accordance with the manufacturer's guidelines (ACDBio, United States of America). Briefly, FFPE sections were incubated for an hour at 60°C and dehydrated via two 5‐min incubations in xylene solutions, followed by two 1‐min incubations in 100% ethanol. Afterwards, tissues underwent a series of hydrogen peroxide blocking (10 min at RT), target antigen retrieval with the RNAscope Co‐detection Target Retrieval Solution (ACDBio #323166) for 30 min at 40°C, and protease treatment with the RNAScope Protease Plus reagent (ACDBio #322331) for 30 min at 40°C. Channel‐2 probes against human *SPP1* (ACDBio #420101‐C2) were applied at 1:50 dilution in the ACD Probe Diluent Reagent for 2 h at 40°C. An extra sample was included which was hybridized with negative control probes (ACDBio #320871). Sections were washed with 1× wash buffer in between every amplification step. For the detection of the SPP1 probe, the HRP‐C2 signal was developed by incubating with the Opal690 dye for 30 min at 40°C, after which an HRP‐blocker was applied to the slide to block further signal amplification. All incubation periods were done in the HybEz hybridization system (ACDBio). A similar hybridization step was applied to the slide designated as the negative control. Slides were washed with 1× RNAscope wash buffer (ACDBio, #310091) in between each step.

Slides were subject to immunofluorescence following in situ hybridization. Tissue sections were incubated for 30 min at room temperature with a blocking solution of 10% normal donkey serum and 0.1% BSA in phosphate buffered saline (PBS), and then overnight at 4°C in a 0.1% BSA‐PBS solution containing primary antibodies against IBA1 (rabbit polyclonal, WAKO, #019–19,741; 1:200) and Aβ (mouse monoclonal, clone 6F/3D, DAKO, #MO872; 1:100). The negative control was incubated overnight with 100 μL PBS‐0.1% BSA. The following day, after washing in a 0.1% Tween20‐PBS solution (PBST), samples were incubated in secondary antibody solutions (1:400 donkey anti‐mouse AlexaFluor488, Thermofisher #A21202, and 1:400 donkey anti‐rabbit AlexaFluor594, Thermofisher #A21207, in PBST) for 2 h at room temperature. Lastly, slides were incubated with DAPI to stain for nuclei, and then coverslipped with ProLong Glass mounting medium (Thermo Fisher Scientific, Hampton, NH) and stored at 4°C in the dark prior to imaging. Slides were washed with PBST in between steps during immunofluorescence.

#### 2.4.2 Imaging

Microscopy images were acquired at 40× magnification using the Leica TCS SP8 X White Light Laser Confocal Microscope (Leica Microsystems). We identified regions of interest (ROI) based on the IBA1 and Aβ signal and imaged six ROIs for samples from AD donors (three with plaques and three without). Three non‐plaque ROIs were imaged from samples from control donors, as no amyloid plaques were detected. We took z‐stacks in 0.2 μm step sizes, resulting in stack sizes between 2 and 5 μm. Images were acquired from the gray matter, with ROIs being selected close to the edge of the tissue to ensure we were not imaging from the white matter.

#### 2.4.3 Image Analysis

Image analysis was performed using a combination of ImageJ and QuPath. Images were first imported into ImageJ, and z‐stacks were summarized based on the maximum signal intensity. In line with previous reports (Mabrouk et al. 2022), DAPI stained some Aβ plaques. To improve nuclear segmentation, we also pre‐processed the maximum projection images to remove Aβ plaques from the DAPI channel. This was done by creating masks from the Aβ channel, converting the signal to 8‐bit format, and using a signal threshold of 99–255. These masks were then removed from the corresponding DAPI channel, and the resulting image was exported for analysis on QuPath.

We segmented cells in QuPath based on the integrated CellPose extension (Stringer et al. 2021). This was done by using the ‘cyto’ pretrained model. We then detected and quantified *SPP1* probes within segmented nuclei based on the subcellular detection plugin in QuPath using a minimum spot size of 5 μm, determined by measuring the mean area of single spots. For clustered *SPP1* signals, the number of dots was estimated by QuPath based on a minimum and maximum spot size of 5–6 μm, respectively. Nuclei were classified as *SPP1*‐positive based on an intensity threshold of 50, which was determined using the signal from the negative control sample. To provide a semi‐quantitative analysis of *SPP1* expression, we followed the recommendations from ACDbio to calculate a histology score (H‐score) for each donor (Jolly et al. 2019; Sari et al. 2025). The H‐score is a semi‐quantitative scoring method, in which cells are classified into 5 bins according to the number of detected dots, with bin0 having < 1 dot, bin1 having 1–3 dots, bin2 having 4–9 dots, bin3 having 10–15 dots, and bin4 having > 15 dots. Cells are then summarized per sample with the formula:

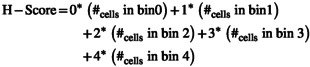




An increase in the H‐score indicates that a larger proportion of cells within the tissue sample are showing positive staining for the marker of interest.

We also used the “Create composite classifier” function in QuPath to classify nuclei as myeloid cells based on the mean intensity of the IBA1 signal. Due to the varying autofluorescence of each human tissue brain sample, a classifier was developed for each donor and applied consistently across all analyzed regions. Detection specificity of the QuPath classifiers was validated by applying the thresholds to negative control images, ensuring no background noise was detected. Lastly, in the AD plaque‐positive ROIs, we annotated Aβ plaques and measured their distance to nuclei using the QuPath function “Distance to 2D annotations.”

#### 2.4.4 Statistics

Statistical analysis was performed using R v4.4.1. After checking for normality using the Shapiro–Wilk and differences in variance using the Bartlett test, we compared the number of IBA1+ nuclei, *SPP1*+/IBA1+ nuclei, and H‐scores between donors in each diagnosis group using either the Welch two‐sample *t*‐test (for normally distributed data) or the Wilcoxon rank‐sum test (for non‐normally distributed data).

## 3 Results

### 3.1 Generating a Complementary snRNAseq Dataset to Study the Cellular Interactome in Alzheimer's Disease

**FIGURE 1:**
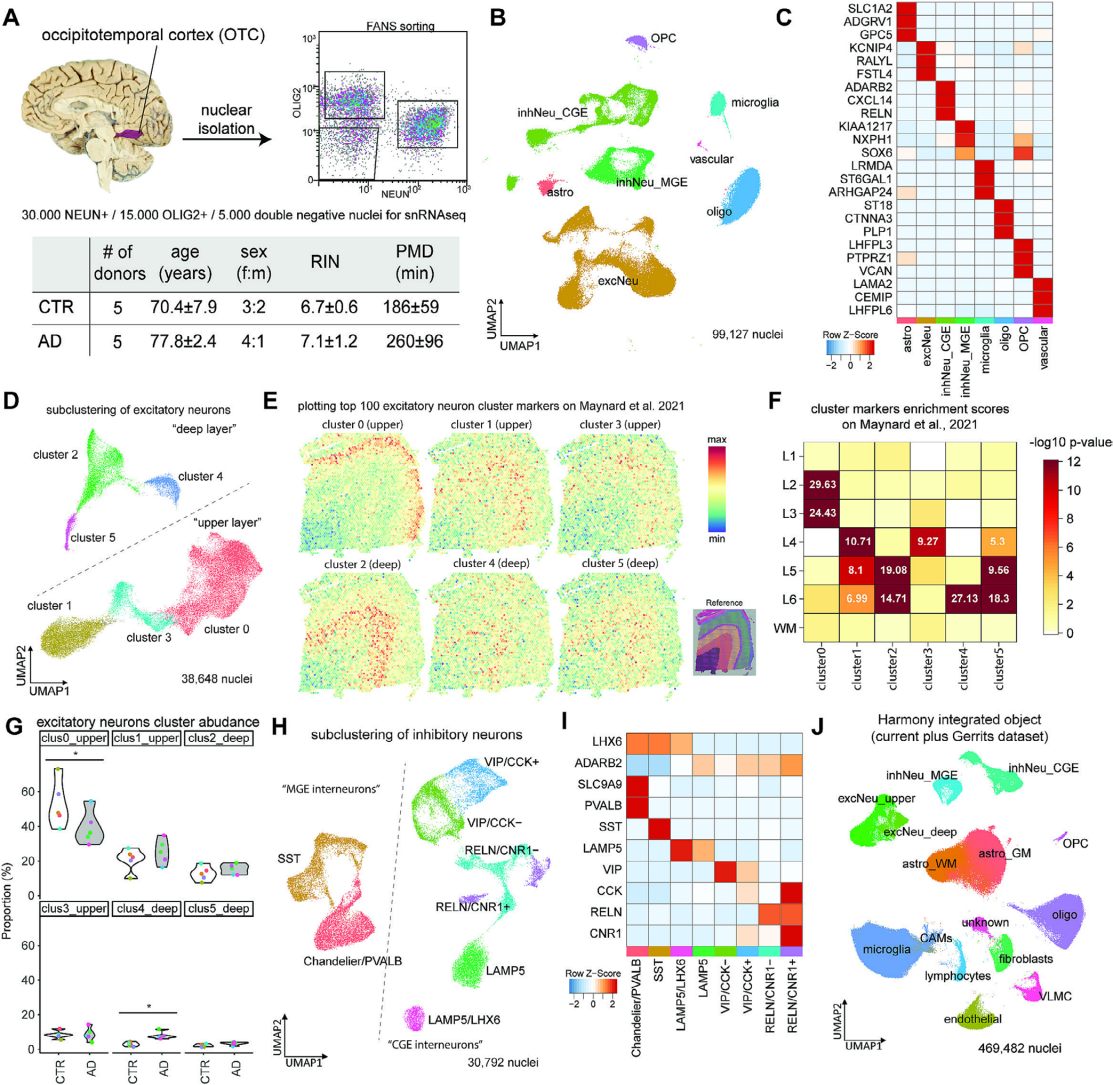
Generating a complementary dataset to study the interactome in Alzheimer's disease. (A) Study design: Single nucleus transcriptomes from 10 occipitotemporal cortex samples were generated from fresh frozen tissues obtained from control (CTR, *n* = 5) and Alzheimer's disease (AD, *n* = 5) donors. These tissues were previously used in (Gerrits et al. 2021). Samples were enriched for neuronal and oligodendrocyte lineage nuclei by fluorescence activated nuclear sorting (FANS). (B) UMAP depicting the annotated cell types from *n* = 99,127 nuclei in the dataset. (C) Heatmap depicting the top 3 identified protein‐coding marker genes per cluster, which were used for cell type annotation. Differentially expressed genes are listed in Table S2. (D) UMAP depicting clusters identified within the 38,648 nuclei annotated as excitatory neurons. Clusters were classified as upper or deep layer excitatory neurons. (E) Spatial plots used to aid annotation of each excitatory neuronal cluster, depicting module scores of the top 100 markers of each cluster in a previously published on a previously spatial transcriptomics dataset (Maynard et al. 2021). Inset shows the cortical layers identified in the original publication. (F) Enrichment scores of the top 100 markers from each excitatory neuronal cluster in spatially defined clusters (see inset of Figure 1E) of a published spatial transcriptomics dataset. (G) Analysis of the association of diagnosis and the relative abundance of each excitatory neuronal cluster. Statistical analysis was performed using Propeller by constructing a linear model with diagnosis as the independent variable with sex, age, and postmortem delay as covariates. *, FDR‐corrected *p* value < 0.05. Each dot depicts one sample. (H) UMAP depicting clusters identified within the 30,792 nuclei annotated as inhibitory neurons. (I) Heatmap depicting the top marker genes per cluster, which were used to aid cell type annotation. A full list of the results from the differential gene expression analysis is available in Table S3. Markers were identified using the FindMarkers function in Seurat, using a two‐tailed Wilcoxon test with an FDR‐corrected *p* value threshold of 0.05. J. UMAP depicting the annotated cell types from the joint analysis of *n* = 469,482 nuclei after integrating the current dataset generated in Figure 1A with the glia‐enriched dataset reported in (Gerrits et al. 2021). Abbreviations: Astro_GM, gray matter astrocytes; Astro_WM, white matter astrocytes; CAMs, CNS‐associated macrophages; Inh_CGE, inhibitory neurons from caudal ganglionic eminence; Inh_MGE, inhibitory neurons from medial ganglionic eminence; OPC, oligodendrocyte precursor cells; RIN, RNA integrity number; PMD, postmortem delay; VLMC, vascular leptomeningeal cells.

To complement our previously generated snRNAseq dataset, which was enriched for NEUN^−^/OLIG2^−^ nuclei (Gerrits et al. 2021), we generated a neuron‐ and oligodendrocyte‐enriched snRNAseq dataset. This dataset was generated from 5 CTR and 5 AD samples also used in the Gerrits et al. (2021) study (Figure 1A, Table S1). This resulted in 99,127 nuclei, which we could cluster and annotate into the major CNS cell types (Figure 1B) based on their expression of known marker genes (Figure 1C, Table S2). Most of our nuclei were annotated as neuronal (39%) or oligodendrocytic (22.7%) (Figure S1A). Neurons (identified based on *SNAP25* expression) were separated into excitatory (*SLC17A7* expressing, *n* = 38,648) or inhibitory (*GAD2* expressing, *n* = 30,792) neurons. In line with inhibitory neuron taxonomy literature (Hodge et al. 2019), we further differentiated inhibitory neurons as originating from the medial (MGE, *n* = 18,614) or caudal ganglionic eminence (CGE, *n* = 12,178), based on expression of *LHX6* and *ADARB2*, respectively. Oligodendrocyte lineage cells were differentiated as mature oligodendrocytes (*PLP1*/*MBP* expressing, *n* = 19,946) and oligodendrocyte precursor cells (OPCs, *PDGFRA*/*BCAN* expressing, *n* = 3572). While smaller in their relative contribution, we could also identify astrocytes (*SLC1A2/AQP4* expressing, *n* = 2553), microglia (*CSF1R*, *P2RY12* expressing, *n* = 3378), and vascular cells (*PECAM1*/*CLDN5/PDGFRB/DCN*, *n* = 238). In line with previous reports (Tasic et al. 2018), neurons had a higher overall RNA content compared to other cell types in this dataset (Figure S1B).

To annotate the neuronal nuclei, we subclustered the excitatory and inhibitory neurons separately. In the excitatory neurons, we identified six neuronal clusters (Figure 1D). These clusters were annotated by calculating marker genes per cluster (Table S3) and investigating the enrichment of each cluster's markers in a previously published spatial transcriptomics cortical dataset (Maynard et al. 2021). By comparing module scores for each cluster's top 100 markers, we observed that our excitatory neuronal clusters reflected upper and deep layer neurons (Figure 1E). This was corroborated by treating the top 100 markers as gene sets and calculating their enrichment per cluster (Figure 1F, Table S4). When comparing the relative abundance of the excitatory neurons (Figure 1G, Table S5), in AD donors we observed a significant decrease in the relative proportion of cluster 0 (layer 2/3) excitatory neurons, as well as an increase in the relative proportion of cluster 4 (layer 6) excitatory neurons. This is in line with reports of increased vulnerability of superficial neuronal layers to degeneration in AD (Romito‐DiGiacomo et al. 2007).

We similarly subclustered the inhibitory neurons (Figure 1H), which were classified based on known molecular markers (Figure 1I, (Hodge et al. 2019; Lin et al. 2025)) and the marker genes they were enriched for (Table S3). Within the *LHX6*‐expressing MGE interneurons, we identified a group of *SST*+ cells, as well as *SLC9A9‐expressing* chandelier cells. In line with previous reports (Taniguchi et al. 2013), we also detected an enrichment of *PVALB* expression within the chandelier cells. CGE interneurons were separated based on their expression of marker genes such as *LAMP5*, *VIP*, *CCK*, *RELN*, and *CNR1* (Table S3). We also identified a population of *LAMP5*‐expressing CGE interneurons that expressed MGE marker *LHX6* (Hodge et al. 2019; Wei et al. 2022). We did not detect differences in the relative proportions of inhibitory neuron subtypes between control and AD samples (Table S5).

To explore potential altered cell–cell communications in AD, we integrated our dataset with the dataset previously reported in (Gerrits et al. 2021) using Harmony (Korsunsky et al. 2019). This resulted in an object with 469,482 nuclei (Figure S1C). Based on marker gene expression (Figure S1D, Table S6), we could annotate all the expected cell types in this integrated dataset (Figure 1J). This included gray‐ (70,918 nuclei) and white‐matter (39,854 nuclei) astrocytes, CNS‐associated macrophages (CAMs, 1408 nuclei), endothelial cells (23,295 nuclei), upper (29,678 nuclei) and lower layer (9216 nuclei) excitatory neurons, fibroblasts (17,582 nuclei), CGE (21,253 nuclei) and MGE (12,818 nuclei) interneurons, lymphocytes (9244 nuclei), microglia (158,836 nuclei), oligodendrocytes (52,118 nuclei), OPCs (2813 nuclei), vascular leptomeningeal cells (VLMCs, 10,801 nuclei), and a previously reported (Gerrits et al. 2021) cluster of unknown cells (9648 nuclei).

### 3.2 Microglia–Microglia SPP1 Signaling is Predicted to be Increased in AD

**FIGURE 2:**
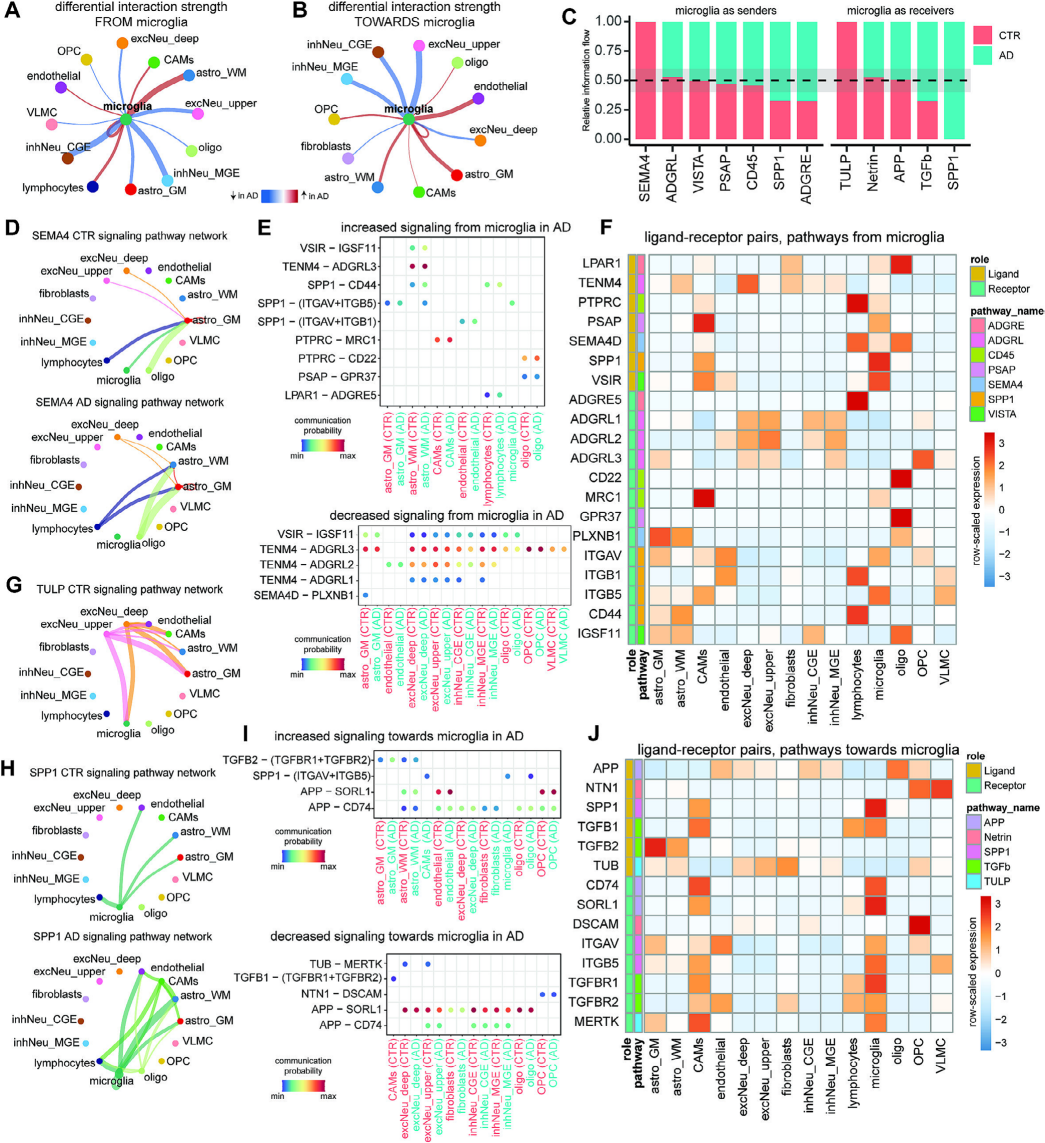
Microglia–microglia SPP1 signaling is predicted to be increased in AD. (A) Predicted differences in cell–cell signaling probability originating from microglia between control and AD donors, as calculated in CellChat. (B) Predicted differences in cell–cell signaling probability directed towards microglia between control and AD donors, as calculated in CellChat. Line thickness in Figure 2A,B corresponds to the strength of differential predicted signaling, with red indicating increased predicted signaling probability in AD, and blue indicating increased signaling probability in controls. Both plots show how microglia–microglia interactions are increased in AD samples. (C) Relative predicted information flow, representing a summary of all communication probabilities, for signaling pathways in control and AD donors that originate from (left) or target (right) microglia. A relative flow of 0.5 (dashed line) would indicate equal detection of a given pathway in both control and AD samples. Gray box depicts y‐axis values between 0.4 and 0.6. (D) Cell–cell communication between cell types in the context of SEMA4 signaling, which microglia are predicted to contribute to in control but not AD samples. (E) Dot plot depicting the predicted communication probabilities of various signaling pathways originating from microglia that were increased (top) or decreased (bottom) in AD versus control donors. (F) Heatmap depicting the average expression of ligands and receptors indicated in Figure 2E in each cell type in the dataset. Annotation bars indicate the pathway each gene is involved in, and whether it acts as a ligand or receptor in said pathway. (G) Cell–cell communication between cell types in the context of TULP signaling, which originates from excitatory neurons towards microglia and is found exclusively in control samples. (H) Cell–cell communication between cell types in the context of SPP1 signaling, in which microglia–microglia signaling is predicted in AD but not control contexts. (I) Dot plot depicting the predicted communication probabilities of various signaling pathways targeting microglia that were increased (top) or decreased (bottom) in AD versus control donors. (J) Heatmap depicting the average expression of ligands and receptors indicated in Figure 2I in each cell type in the dataset. Annotation bars indicate the pathway each gene is involved in, and whether it acts as a ligand or receptor in said pathway. Lines in Figure 2D,G,H connect a source (ligand‐expressing) and a target (receptor‐expressing) cell, with line widths correspond to the predicted interaction strength, and line colors depicting the predicted source cell type in each given interaction. Abbreviations: Astro_GM, gray matter astrocytes; Astro_WM, white matter astrocytes; CAMs, CNS‐associated macrophages; Inh_CGE, inhibitory neurons from caudal ganglionic eminence; Inh_MGE, inhibitory neurons from medial ganglionic eminence; OPC, oligodendrocyte precursor cells; VLMC, vascular leptomeningeal cells.

In the integrated dataset, we calculated inferred changes in cell–cell communication using CellChat (Jin et al. 2021, 2025) (Table S7). We observed decreased communication between neuronal subtypes in AD vs. control donors (Figure S2A), evident from the loss of scores for pathways described to be related to synapse assembly such as neurexin, ephrin, neural cell adhesion molecule, laminin, and latrophilin (Bartas et al. 2025; Rodin et al. 2020; Araç and Li 2019) (Figure S2B). Focusing on microglia‐relevant pathways, we detected AD‐related alterations in the communication of CNS cell types that either receive information from (Figure 2A) or send information towards (Figure 2B) microglia. In AD donors, microglia are predicted to have stronger interactions with CAMs, endothelial cells, lymphocytes, and white matter astrocytes, while receiving stronger signals from both astrocyte groups, CAMs, endothelial cells, oligodendrocytes, and OPCs. Notably, we also observed increased cell–cell interactions between microglia in AD.

To better understand the nature of these differential interactions, we investigated the pathways microglia were involved in, either as senders or receivers of signals (Figure 2C). CellChat predicted microglia in our dataset to be senders (i.e., ligand‐expressing) in seven pathways (SEMA4, ADGRL, VISTA, PSAP, CD45, SPP1, and ADGRE), and receivers (i.e., receptor‐expressing) in five pathways, (TULP, Netrin, APP, TGFβ, and SPP1). Pathways where relative information flow to be above 0.6 (stronger in CTR) or below 0.4 (stronger in AD) were considered relevant for disease. From these pathways, we first focused on those that were uniquely observed in either control or AD subsets.

Of the pathways where microglia were senders, only SEMA4 signaling was unique to a diagnosis group and only detected in control samples. Microglia were predicted to take part in this pathway signaling to gray matter astrocytes (Figure 2D). Specifically, microglia expressed *SEMA4D* that targets *PLXNB1* receptors on gray matter astrocytes (Figure 2E,F). PLXNB1 has previously been described as relevant in plaque‐associated astrocytes in a mouse model of AD, where deletion of *Plxnb1* was shown to increase the abundance of disease‐associated microglia (Huang et al. 2024). There were two pathways in which microglia were receivers in a specific diagnosis group. These were the TULP pathway (Figure 2G), which was uniquely detected in microglia from control donors, and the SPP1 pathway (Figure 2H), which was uniquely detected in microglia from AD donors. The TULP pathway (ligand *TUB*—Tubby Bipartite Transcription Factor) was predicted to originate from both groups of excitatory neurons to target endothelial cells, CAMs, astrocytes, and microglia in the control donors (Figure 2G). This pathway was undetected in AD samples. Microglial involvement in this pathway was predicted to occur via the activation of the *MERTK* receptor by the *TUB* ligand (Figure 2I,J). This pathway has previously been described to be an ‘eat‐me’ signal (Caberoy et al. 2010), and its loss in AD presumably reflects the neuronal loss observed in the disease.

SPP1 signaling towards microglia was uniquely observed in AD, with predicted ligands originating from CAMs, oligodendrocytes, and microglia (Figure 2J). This suggests that SPP1 signaling contributes to the increased microglia–microglia signaling observed in Figure 2A,B. This signaling pathway is not unique to AD, although SPP1 signaling from CAMs and oligodendrocytes was not observed in control samples. In nuclei from control donors, microglia were predicted to send *SPP1* to both gray and white matter astrocytes, endothelial cells, and lymphocytes. The strengths of all these interactions were predicted to increase in AD, through the *CD44* receptor for white matter astrocytes and lymphocytes, and a combination of different integrin receptors (*ITGAV* and either *ITGB5* or *ITGB1*) for gray matter astrocytes and endothelial cells (Figure 2E,F). Microglial targeting by *SPP1* was predicted to occur via *ITGAV* and *ITGB5* receptor activation (Figure 2I), receptors they expressed (Figure 2J).

Regarding pathways with altered signaling strength between both diagnosis groups, we observed an AD‐associated increase in ADGRE and TGFβ signaling (Figure S2C). ADGRE signaling mainly involved microglial expression of the ligand *LPAR1*, targeting the *ADGRE5* receptor on lymphocytes (Figure 2E,F). Additionally, the overall increased TGFβ signaling towards microglia (which expressed the *TGFBR1* and *TGFBR2* receptors) was mediated by *TGFB2* signaling from astrocytes. Notably, we also observed a loss of signaling from CAMs (which express *TGFB1* ligand) towards microglia in AD.

Other pathways that were detected in our CellChat analysis included the ADGRL, VISTA, PSAP, and CD45 pathways, where microglia were predicted to participate by expressing the ligand (Figure 2C,E,F). The ADGRL pathway was mainly mediated by *TENM4* ligand expression in microglia, via the *ADGRL1*, *ADGRL2*, and *ADGRL3* ligands expressed by astrocytes, endothelial cells, neurons, oligodendrocytes, and VLMCs. VISTA signaling from microglia (via the *VSIR* ligand) was predicted to target different cell types via the *IGSF11* receptor, including astrocytes, excitatory neurons, inhibitory neurons, and oligodendrocytes. PSAP signaling involved microglial expression of the ligand *PSAP* that interacts with the receptor *GPR37*, expressed by oligodendrocytes. CD45 signaling involved microglial expression of the *PTPRC* ligand, towards either CAMs (via the *MRC1*) or oligodendrocytes (via *CD22*).

Regarding pathways with microglial involvement as receivers (Figure 2C,I,J), we observed Netrin signaling in both datasets, based on *NTN1* gene expression in OPCs and *DSCAM* expression in microglia. We also observed APP signaling towards microglia (via either the *SORL1* or *CD74* receptors). Regarding APP signaling, microglia were predicted to receive differential signaling depending on the cell types. Notably, neuronal APP signaling towards microglia was mostly decreased, which we hypothesize reflected neuronal loss.

In summary, using CellChat, we were able to predict how CNS cell interactions were altered in AD. We identified several microglia‐related signaling changes, of which the most striking was a microglia–microglia SPP1 signaling pathway uniquely detected in AD donors.

### 3.3 Microglia–Microglia SPP1 Signaling in AD

**FIGURE 3:**
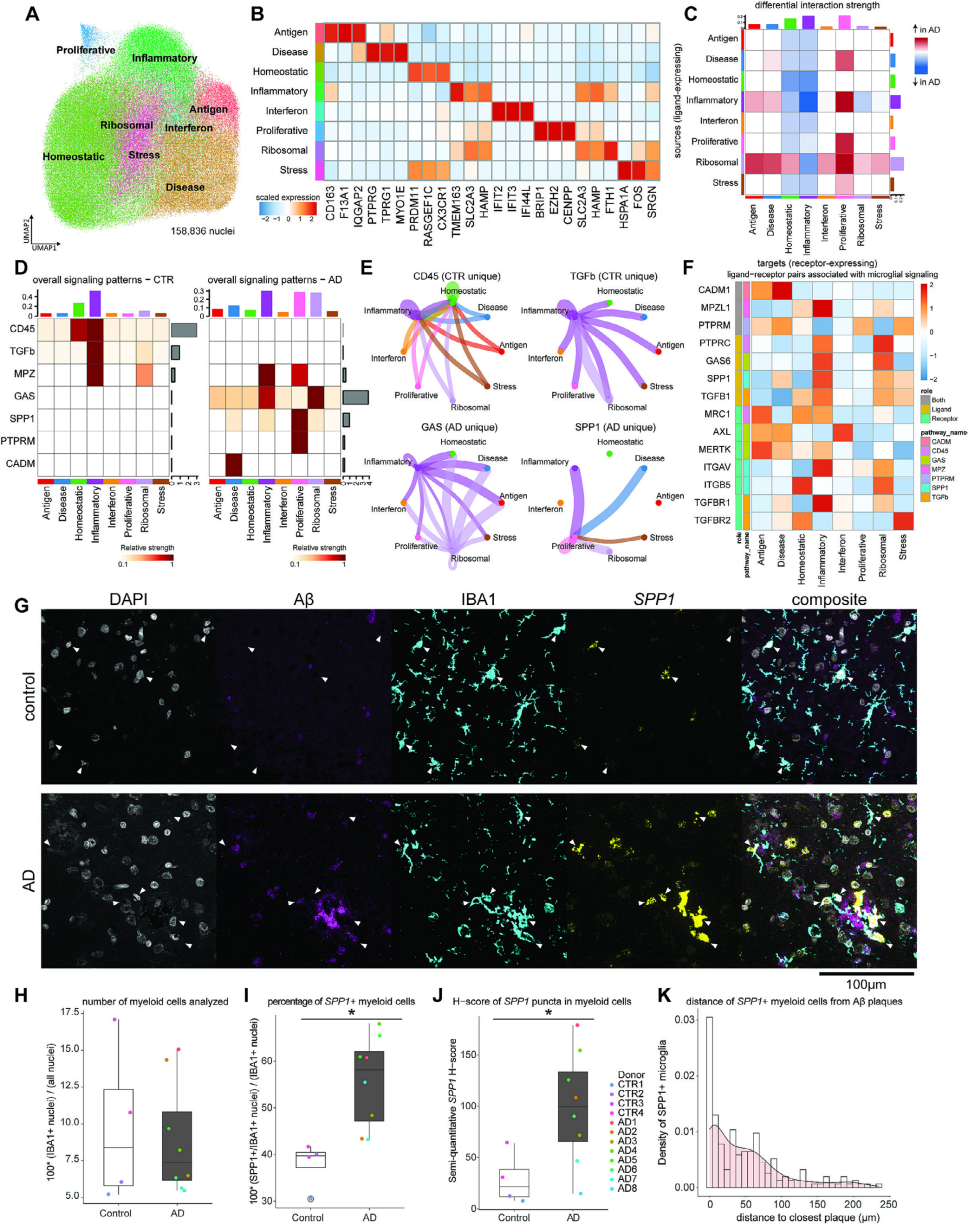
Microglia–microglia SPP1 signaling in AD. (A) UMAP depicting the clustering of *n* = 158,836 microglial nuclei into eight states within the integrated dataset. (B) Heatmap depicting the top 3 identified protein‐coding marker genes per microglial state, which were used for annotation. Differentially expressed genes are listed in Table S8. (C) Heatmap depicting differences in predicted total cell–cell communication strength between microglial states in control and AD donor groups. Microglial states on the y‐axis are the senders (i.e., ligand‐expressing), while microglial states on the x‐axis are the receivers (i.e., receptor‐expressing). Colors represent increased predicted cell–cell communication in nuclei from AD (red) or control (blue) donors. (D) Overall involvement (i.e., aggregated outgoing or incoming signaling) of each microglial state in the control (left) and AD (right) data. (E) Cell–cell communication within microglial states predicted by cell chat. Chord diagrams depict pathways uniquely identified in microglia from control (CD45, TGFβ, top) and AD (GAS, SPP1) microglia. Lines connect a source (ligand‐expressing) and a target (receptor‐expressing) state, with line widths correspond to the predicted interaction strength, and line colors depicting the predicted source state in each given interaction. (F) Heatmap depicting the average expression of ligands and receptors identified in the microglial CellChat analysis within each microglial state. Annotation bars indicate the pathway each gene is involved in, and whether it acts as a ligand receptor, or both in each pathway. (G) Representative images of DAPI, Aβ, IBA1, and SPP1 signals acquired from FFPE samples from a control and AD donor. Images are maximum projection images of a z‐stack acquired using confocal microscopy. (H) Box plot depicting the number of IBA1+ nuclei in the analyzed fields of view between diagnosis groups. (I) Box plot depicting the number of *SPP1*+/IBA1+ nuclei in the analyzed fields of view between diagnosis groups. (J) Box plot depicting a semi‐quantitative histology score (H‐score) for *SPP1* expression in IBA1+ nuclei per sample. Points in panels H‐J are colored by sample. (K) Histogram depicting the frequency of *SPP1*+/IBA1+ nuclei in the analyzed fields of view within AD samples, stratified by the distance to the closest Aβ plaque. Shaded line plot depicts the density estimated as calculated with the geom_density function in ggplot2. *, *p* < 0.05 in the Welch two‐sample *t*‐test; boxplot borders correspond to the 25th and 75th percentile, whereas whiskers are drawn from each border +/− 1.5× interquartile range. Data points exceeding the 1.5*interquartile range are depicted with a black outline. Abbreviations: Antigen, antigen‐presenting microglia; Disease, disease‐associated microglia; Interferon, interferon‐responsive microglia; Ribosomal, ribosomal‐responsive microglia; Stress, stress‐responsive microglia.

To further explore how microglia–microglia SPP1 signaling might be altered in AD, we repeated the CellChat analysis within microglia only. We first sub‐clustered the 158,836 nuclei annotated as microglia from the integrated object, identifying eight microglia transcriptional states based on their marker genes (Figure 3A,B, Table S8). These states included homeostatic microglia (*PRDM11, RASGEF1C, CX3CR1* enriched, *n* = 74,602), disease‐associated microglia (*PTPRG*, *TPRG1*, *MYO1E* enriched *n* = 28,682), inflammatory microglia (*TMEM163*, *SLC2A3*, *HAMP* enriched, *n* = 23,704), stress‐responsive microglia (*HSPA1A*, *FOS*, *SRGN* enriched, *n* = 15,259), antigen‐presenting microglia (*CD163*, *F13A1*, *IQGAP2* enriched, *n* = 11,050), interferon‐responsive microglia (*IFIT2*, *IFIT3*, *IFI44L* enriched, *n* = 3341), proliferative microglia (*BRIP1*, *EZH2*, *CENPP*, *n* = 1806) and ribosomal‐responsive microglia (*SLC2A3*, *HAMP*, *FTH* enriched, *n* = 392). The annotation of these states was further supported by the expression of distinct microglial signatures previously observed in iPSC‐derived microglia xenografted into an amyloidogenic environment (Mancuso et al. 2024) (Figure S2D). We calculated cell–cell communication probabilities within this re‐analyzed dataset (Figure 3C), identifying seven pathways between microglia (Figure 3D, Figure S2E, Table S9): CD45, TGFβ, MPZ, GAS, SPP1, PTPRM, and CADM.

As with the analysis of major cell types, we detected a predicted increase in microglia–microglia SPP1 signaling uniquely in AD (Figure 3D). This prediction was based on expression of *SPP1* ligand by disease‐associated, inflammatory, stress‐responsive, proliferative, and ribosomal‐responsive microglia, signaling towards proliferative microglia (Figure 3E), and expression of the receptors *ITGAV*‐*ITGB5* (Figure 3F). The difference in SPP1‐associated signaling was further supported by investigating the expression of genes associated in this pathway within the microglial states stratified by diagnosis status (Figure S2F,G).

Another pathway uniquely identified in AD microglia was the GAS pathway (Figure 3D), mediated by the expression of the ligand *GAS6* in inflammatory and ribosomal‐responsive microglia, and directed towards all microglial states (Figure 3E), via the *AXL* receptor (Figure S2G). This pathway has been implicated in phagocytosis (Tondo et al. 2019), and, together with evidence that SPP1 signaling promotes microglial phagocytosis (De Schepper et al. 2023), supports previous reports of increased phagocytic microglial profiles in AD (Brown et al. 2026). In our data, increased CADM1‐CADM1 interactions were predicted between disease‐associated microglia, while increased PTPRM‐PTPRM interactions were predicted within the proliferative microglia (Figure S2H, Figure 3F).

Two pathways, CD45 and TGFβ, were uniquely identified in microglia from control donors (Figure 3D,E). CD45 signaling was predicted based on the PTPRC ligand, expressed by all microglial clusters and MRC1 receptor on the homeostatic and inflammatory microglial states (Figure 3F), none of which were detected in AD microglia (Figure S2I). Similarly, TGFβ signaling, mainly mediated by the expression of the *TGFB1* ligand in inflammatory microglia towards TGFBR1 or TGFBR2 receptor expression in the rest of the microglial states, was undetected in microglia from AD donors (Figures 3F and S2I). Lastly, in MPZ signaling, which was identified in both diagnosis groups, *MPZL1* acts as both ligand and receptor (Figure 3F). This signaling pathway was increased in AD between proliferative microglia, and bidirectionally between inflammatory and proliferative microglia (Figure S2H). Concurrently, this signaling pathway is also predicted to be increased in control donors between inflammatory microglia, and bidirectionally between inflammatory and ribosomal‐responsive microglia (Figure S2I).

In summary, our CellChat analyses highlight several microglia–microglia communication pathways that are uniquely present or absent in microglia from AD donors compared to control donors. Several of these pathways have a demonstrated association with phagocytosis.

### 3.4 *SPP1* ‐Expressing Myeloid Cells Are Increased in the AD Brain

The relevance of *SPP1* expression in microglia was analyzed in a separate cohort of AD brain samples. As SPP1/osteopontin is a secreted protein (Lin et al. 2023), we used a combination of RNAscope and immunofluorescence to detect *SPP1* expression in IBA1 expressing myeloid cells in control and AD tissue (Figure 3G, Table S10). After confirming that a similar number of IBA1+ nuclei were imaged between experimental groups (Figure 3H), we compared the number of *SPP1*+/IBA1+ nuclei identified and observed that samples from AD donors had increased numbers of double‐positive nuclei (Figure 3I). We also calculated an H‐score per donor, a semi‐quantitative approach to score probe expression within samples based on grouping nuclei from IBA1+ nuclei into five bins stratified by the amount of RNA molecules detected within them (Jolly et al. 2019; Sari et al. 2025). Using this metric, we observed that IBA1+ nuclei in AD donors had a higher H‐score for *SPP1* compared to controls (Figure 3J). To explore a relationship between *SPP1*‐expression and pathology, we calculated Euclidean distances between IBA1+ nuclei and the nearest Aβ plaques. We observed that the density of *SPP1*+/IBA1+ nuclei was highest near Aβ plaques (Figure 3I, 3K).

## 4 Discussion

Here we generated a snRNAseq dataset enriched for nuclei from neuronal‐ and oligodendrocyte‐lineage cells using a subset of the samples reported in (Gerrits et al. 2021). After integrating both snRNAseq datasets and performing bioinformatic analyses to predict cell–cell communication, we observed increased microglia–microglia communication in AD and identified microglia–microglia SPP1 signaling as a potentially important signaling pathway in AD. Using combined RNAscope and immunofluorescence, we observed that SPP1‐expressing IBA1‐positive cells were increased in AD, and that the number of SPP1‐expressing IBA1+ cells was highest around Aβ plaques.

### 4.1 Microglia as a Player in Cell–Cell Communication in the Context of AD

Microglia perform a variety of important immune and non‐immune functions in conjunction with their surrounding cells. Over the past decade, it has become evident that microglia are relevant in AD pathophysiology (Heneka et al. 2025, 2015). Beyond observations that microglia occupy specific spatial domains, with respect to neuropathological features such as amyloid plaques (Heneka et al. 2015; Orre et al. 2014), there is a growing body of work (e.g., using single cell transcriptomics (Srinivasan et al. 2020; Sun et al. 2023; Gerrits et al. 2021), as well as proteomics (Mrdjen et al. 2025)) reporting on the distinct states microglia adopt in AD. For instance, isolation and subsequent transcriptomic profiling of microglia around plaques has led to the identification of plaque‐associated signatures (Wood et al. 2022), which contain many genes also reported in other CNS diseases (Paolicelli et al. 2022).

Perturbed signaling between cells in pathological microenvironments likely influences disease progression. For instance, microglial cytokine release in an in vitro model can induce neurotoxic phenotypes in astrocytes (Liddelow et al. 2017), while factors released by astrocytes such as IL33, SFRP1, and complement C3 can drive inflammatory or phagocytic processes in microglia in various models (Vainchtein et al. 2018; Rueda‐Carrasco et al. 2021; Costa et al. 2017). We aimed to use our current single‐nucleus transcriptomic data to identify which known communication pathways might have relevance for AD. While this approach has previously been used in AD, current predictions have focused on neuron‐related signaling (Bartas et al. 2025), possibly due to the relatively low number of microglia obtained in unsorted single cell/nucleus libraries. While our analysis also predicts reduced neuronal signaling, especially regarding pathways known to be relevant for synapse formation and maintenance, we were able to investigate altered signaling between glial populations in better detail due to our depletion strategy of NEUN+/OLIG2+ nuclei in the Gerrits et al. (2021) dataset.

Our data predict two pathways between microglia and other cell types to be lost in AD. The first is the TULP pathway, based on the expression of *TUB* in both excitatory neuron groups. This pathway has been reported to regulate microglial signaling via the MerTK pathway in mice, and is characterized as an eat‐me signal important in CNS homeostasis (Caberoy et al. 2010). This signaling pathway, predicted to activate receptors expressed on phagocytes such as astrocytes, microglia, and CAMs, was fully absent in AD donors. This observation might be caused by the neuronal loss observed in AD. Another pathway predicted to be lost in AD relates to semaphorin‐4 signaling, based on *SEMA4* signaling from microglia towards the *PLXNB1* receptor in gray matter astrocytes. Astrocytic Plexin‐B1 signaling has been implicated in the regulation of the proximity of both astrocytes and microglia around amyloid‐beta plaques in an amyloidogenic mouse model (Huang et al. 2024), and its deletion in murine and human iPSC‐derived astrocytes was shown to decrease the expression of an astrocytic signature associated with cognitive decline (Comandante‐Lou et al. 2025).

In turn, our data predict increased TGFβ signaling from gray matter astrocytes towards microglia in AD. Microglial TGFβ signaling, typically seen as anti‐inflammatory (Kapoor and Chinnathambi 2023; Butovsky et al. 2014), is important for the maturation of microglia (Spittau et al. 2020), and the maintenance of their homeostatic state (Zöller et al. 2018). While there are reports that associated ligands and receptors are decreased in AD patients (Mocali et al. 2004; Tesseur et al. 2006), its activation has been associated with increased clearance of amyloid‐β (Wyss‐Coray et al. 2001; Kuroda et al. 2020). As such, it has been discussed as a potentially neuroprotective pathway against the neurodegeneration seen in AD (Caraci et al. 2011; Eleni et al. 2025).

We observe two pathways in which microglia have increased participation in AD, the first of which is the ADGRE pathway. This pathway was predicted to be present based on the microglial expression of the LPAR1 ligand towards ADGRE5 receptors in lymphocytes. ADGRE5, also known as CD97, has best been studied in the context of tumor infiltration and has been shown to impact leukocyte trafficking in mouse models (Hamann et al. 2010). Although understudied in the context of AD, there is recent evidence to suggest that lymphocytes also play a role in disease progression and response to medication (Plantone et al. 2022). The increased ADGRE5 signaling we observe might thus lead to the observation of T‐cell trafficking in AD (Ma et al. 2024).

The other microglia‐associated pathway that is increased in AD is SPP1. While we will discuss microglia–microglia SPP1 signaling in the next section, we also observed AD‐specific SPP1 signaling predicted from CAMs and oligodendrocytes towards microglia. The AD‐specific participation of CAMs in SPP1 signaling is in line with the results of a previous study, which found border‐associated macrophages to send out SPP1 signals towards T‐cells and endothelial cells (Kearns et al. 2023). Notably, this signaling has been studied in a mouse model, where CAMs‐derived SPP1 (De Schepper et al. 2023) has been reported to induce phagocytosis of synapses and amyloid‐beta. There is less evidence for disease‐associated expression of *SPP1* in oligodendrocytes, and its expression level was moderate compared to microglia and CAMs. However, oligodendrocytes have notably been reported to express *SPP1* in AD brains (Lopes et al. 2024), and might be another emerging factor in the rising importance of this cell type in AD pathophysiology (Kedia and Simons 2025).

### 4.2 Insights Into the Role of Microglia–Microglia Signaling in AD

In addition to making predictions about how microglia impact other CNS cell types in pathological conditions, our data also allowed us to predict how microglia–microglia interactions might be affected in AD. As mentioned, a wide spectrum of microglial signatures can be detected based on sc−/sn‐RNAseq studies (Srinivasan et al. 2020; Sun et al. 2023; Gerrits et al. 2021; Olah et al. 2020). In these studies, different microglial states, as defined by each signature, were differentially abundant in control and diseased samples. As such, it is intriguing to consider whether altered communication between these different microglial states has disease‐relevant implications.

Our data suggest distinct microglia–microglia communication landscapes in the AD brain, as most detected pathways were unique to either diagnosis group. Only one microglia–microglia signaling pathway (MPZ) was predicted in both control and AD donors. The gene that serves as both ligand and receptor in this pathway, *MPZL1*, has previously been identified as a risk gene for late onset AD, and was also reported to be part of an immune‐expressed gene network enriched in AD (Felsky et al. 2018). Regarding pathways present only in controls, we did not observe CD45 and TGFβ signaling within microglia in AD. TGFβ signaling is important for microglial identity and in particular, microglia–microglia TGFβ signaling is important to retain microglia in a homeostatic state in mice (Bedolla et al. 2024). The loss of TGFβ signaling in AD might represent a potential mechanism for the transition from a healthy, homeostatic to more disease‐associated states. The loss of CD45 signaling would be congruent with this hypothesis, as the depletion of CD45 in either in vitro or mouse models resulted in decreased phagocytic capacity of microglia (Tan et al. 2000; Zhu et al. 2011; Vo et al. 2025).

In turn, microglia increasingly communicated with each other via the CADM, GAS, PTPRM, and SPP1 pathways in AD brains. Two of these pathways were predicted to occur within a microglial state. First, CADM signaling was predicted to take place between disease‐associated microglia. As this gene was reported in the initial mouse study describing disease‐associated microglia (Keren‐Shaul et al. 2017), and noting that cell adhesion processes mediated by this gene have been described in peripheral immune populations (Darlington et al. 2026), it is tempting to consider whether this process plays a role in the clustering of microglia around plaques and the specific disease‐associated signatures induced around this niche (Mallach et al. 2024). Next, PTPRM was predicted to be a signaling pathway relevant specifically within proliferative microglia. While not well studied in the context of microglia, this pathway has been shown to promote cell proliferation in cervical cancer patients with lymph node metastasis (Liu et al. 2020), and might thus represent a possible mechanism for the increased proliferation of microglia observed in AD or models of amyloidosis (Gao et al. 2023; Villacampa et al. 2025).

The two remaining pathways have each been implicated in phagocytosis of Aβ plaques. GAS signaling, predicted in our data to originate from inflammatory and ribosomal microglia towards all other microglial states, involves the interactions of the *GAS6* ligand and the *TYRO*, *AXL*, and *MERTK* receptors. Overexpression of different parts of this interaction has been shown to increase Aβ phagocytosis in mouse models (Owlett et al. 2022; Huang et al. 2021). Beyond phagocytosis, it has been suggested to modulate inflammatory processes in disease (Huang et al. 2021). The other pathway, SPP1, was also predicted in the CellChat analysis that included all cell types, highlighting the prominence of this pathway in our dataset. SPP1 is a secreted molecule that is upregulated in both developmental (Lawrence et al. 2024; Mastenbroek et al. 2024) and injury or diseased contexts.

As a matrix‐associated glycoprotein, SPP1 accumulates in the extracellular matrix and shapes the local microenvironment in multiple pathologies (Yim et al. 2022). For instance, SPP1 has proven relevant in mouse models of brain ischemia (Lan et al. 2024; Li et al. 2025), as well as de−/re‐myelination (Nilsson et al. 2023; Selvaraju et al. 2004). SPP1 is also relevant for AD, where its expression at both RNA and protein level has been associated with increased rates of cognitive decline and disease severity in samples from AD donors (Lopes et al. 2024; Qiu et al. 2023). Notably, SPP1 is part of many previously described disease‐associated microglial gene sets (Paolicelli et al. 2022), including those defined from mouse models of amyloidosis (Keren‐Shaul et al. 2017) and neurodegeneration (Krasemann et al. 2017), as well as in datasets generated from postmortem human samples from donors with AD (Srinivasan et al. 2020; Sun et al. 2023; Gerrits et al. 2021; Olah et al. 2020). *SPP1*‐enriched subsets of microglia have also been reported in other neurodegenerative conditions such as multiple sclerosis (Alsema et al. 2024; Absinta et al. 2021; Lerma‐Martin et al. 2024), frontotemporal dementia (Al‐Dalahmah et al. 2024), and amyotrophic lateral sclerosis (Masrori et al. 2025). Microglia express integrin receptors (Lin et al. 2023), which also makes them potential targets for SPP1. The putative activation of this pathway, together with the predicted activation of GAS signaling, may promote phagocytosis, consistent with findings from preclinical studies. SPP1 activity was shown to be necessary to induce a phagocytic microglial state in amyloidogenic mice (De Schepper et al. 2023). Similarly, the addition of SPP1 protein to organotypic slice cultures from amyloidogenic mice xenografted with human induced pluripotent stem cell derived microglia led to increased clearance of Aβ (Albertini et al. 2025). The same study also suggested that *SPP1* upregulation contributes to the efficacy of the AD drug Lecanemab (Albertini et al. 2025).

In summary, our data represent potential mechanisms for microglial crosstalk with other microglia, as well as other glial cells, during neuropathology and neurodegeneration in AD. However, the temporal dynamics of how these signaling pathways are altered with disease progression in vivo, and the effectiveness and consequences of any such compensatory mechanisms remain to be established. Additionally, these predicted interactions likely need to take place within spatially proximal cells. While this information is lost during snRNAseq data preparation, adaptation of cell–cell communication tools to study emerging spatial transcriptomic platforms (Jin and Ramos 2022) technologies will help in highlighting truly biologically relevant signaling pathways in disease. We anticipate future studies to shed more light on the potential of SPP1 signaling to modulate AD pathophysiology.

## Author Contributions

M.T.L., I.R.H., S.M.K., and B.J.L.E. conceived the project. I.R.H., S.M.K., and B.J.L.E. supervised the project. J.M.K., M.T.L., E.C.D., A.V., and N.B. performed the experiments. J.M.K. and E.C.D. analyzed the data. J.M.K. and E.C.D. wrote the paper with input from all authors.

## Funding

This work was financially supported by Alzheimer Nederland grants WE.03–2025‐06 (to J.M.K.) and WE.03–2020‐03 (to B.J.L.E.), ZonMW grant MODEM 10510032120006 (to B.J.L.E.), and by the De Cock Hadders Foundation (to E.C.D.).

## Disclosure

Permission to reproduce material from other sources: Publication licenses for graphics made with Biorender are uploaded in the submission.

## Ethics Statement

The authors have nothing to report.

## Consent

The authors have nothing to report.

## Conflicts of Interest

The authors declare no conflicts of interest.

## Supporting information


**Figure S1:** Supporting Information figure related to snRNAseq analysis. (A) Bar plots depicting the proportion of neuronal, oligodendrocytic, and all other cell types combined, in each processed sample to confirm the effectiveness of the FANS enrichment performed prior to generating snRNAseq libraries. (B) Quality control plots within the snRNAseq data generated in Figure 1A, grouped by the cell type annotations as shown in Figure 1B. (C) UMAP separately depicting nuclei from the dataset generated in Figure 1A and the glia‐enriched dataset reported in Gerrits et al. (2021) after integration, colored by the clustering annotations in the joint dataset. (D) Heatmap depicting the top 3 identified marker genes per cluster, which were used to aid cell type annotation. A full list of the results from the differential gene expression analysis is available in Table S6. Markers were identified using the FindMarkers function in Seurat, using a two‐tailed Wilcoxon test with an FDR‐corrected *p* value threshold of 0.05. Abbreviations: Astro_GM, gray matter astrocytes; Astro_WM, white matter astrocytes; CAMs, CNS‐associated macrophages; Inh_CGE, inhibitory neurons from caudal ganglionic eminence; Inh_MGE, inhibitory neurons from medial ganglionic eminence; OPC, oligodendrocyte precursor cells; RIN, RNA integrity number; PMD, postmortem delay; VLMC, vascular leptomeningeal cells.

**Figure S2:** Supporting Information figures related to CellChat analysis. (A) Heatmap depicting differences in predicted total cell–cell communication strength between cell types in control and AD donor groups. Cell types on the y‐axis are the senders (i.e., ligand‐expressing), while cell types on the x‐axis are the receivers (i.e., receptor‐expressing). Colors represent increased predicted cell–cell communication in nuclei from AD (red) or control (blue) donors. (B) Plots depicting differences in the extent that specific cell signaling pathways change between AD and controls within different neuronal clusters. The x axis depicts differential (AD—control) outgoing interaction strength (i.e., the specified neuronal group expresses the ligand), and the y axis depicts differential (AD—control) incoming interaction strength (i.e., the specified neuronal groupthat expresses the receptor). Positive values depict signaling pathways increased in AD, negative values depict signaling pathways decreased in AD, versus controls. Highlighted quadrant in blue represents pathways in which neurons both send and receive less information in AD versus controls. (C) Cell–cell communication between cell types in the context of ADGRE and TGFβ signaling. (D) Heatmap depicting the module score expression of the top 25 markers of each microglial cluster described in (Mancuso et al. 2024) within the microglial states in the dataset (x axis). (E) Predicted differences in cell–cell signaling probability between control and AD donors originating from each microglial state, shown in bold font. Line thickness corresponds to the strength of differential predicted signaling, with red indicating increased predicted signaling probability in AD, and blue indicating increased signaling probability in controls. (F) Feature plots showing the expression of SPP1 and its associated receptor genes in the microglial object, split by control (top) or AD donors (bottom). (G) Heatmap depicting the average expression of genes shown in E within each microglial state. (H) Dot plot depicting the predicted communication probabilities of all signaling pathways identified within microglial states that were increased in AD versus control donors. None of these signaling pairs was found in microglia from CTR donors. (I) Dot plot depicting the predicted communication probabilities of all signaling pathways identified within microglial states that were decreased in AD versus control donors. None of these signaling pairs, except MPZL1‐MPZL1, was found in microglia from AD donors. X‐axis labels in (H and I) depict the source microglial state (ligand expressing) towards the target state (receptor expressing). Abbreviations: Antigen, antigen‐presenting microglia; Astro_GM, gray matter astrocytes; Astro_WM, white matter astrocytes; CAMs, CNS‐associated macrophages; Disease, disease‐associated microglia; Inh_CGE, inhibitory neurons from caudal ganglionic eminence; Inh_MGE, inhibitory neurons from medial ganglionic eminence; Interferon, interferon‐responsive mciroglia; OPC, oligodendrocyte precursor cells; Ribosomal, ribosomal‐responsive microglia; Stress, stress‐responsive microglia; VLMC, vascular leptomeningeal cells.

**Table S1:** Sample and donor characteristics for neuron‐ and oligodendrocyte‐enriched dataset.

**Table S2:** Cluster markers for analysis of major cell types in the generated neuron‐ and oligodendrocyte‐enriched dataset.

**Table S3:** Cluster markers for subclustering analysis of excitatory and inhibitory neurons.

**Table S4:** Geneset enrichment results for excitatory neuronal cluster markers on a spatial transcriptomics dataset.

**Table S5:** Propeller proportion analysis results for excitatory and inhibitory neurons.

**Table S6:** Cluster markers for analysis of major cell types after integrating the current dataset with Gerrits et al.

**Table S7:** CellChat communication predictions for pathways when analyzing major cell types.

**Table S8:** Cluster markers for subcluster analysis of microglia.

**Table S9:** CellChat communication predictions for pathways when analyzing microglial states.

**Table S10:** Statistics for RNAscope analysis.

## Data Availability

The snRNAseq data generated in this paper is available on the Gene Expression Omnibus with accession number GSE335667. The reanalyzed Gerrits et al. (2021) dataset is available on the Gene Expression Omnibus with accession number GSE148822. Code associated with snRNAseq clustering and CellChat analyses is available at https://github.com/jmkotah/AD_snRNAseq. Correspondence and requests for materials should be addressed to Bart Eggen (b.j.l.eggen@umcg.nl).
